# Impact of pain on quality of life (QoL) in patients with acromegaly: profound differences in physical and mental aspects of QoL

**DOI:** 10.1007/s12020-026-04566-y

**Published:** 2026-03-09

**Authors:** Lisa Schock, Anna Lena Friedel, Sonja Siegel, Nicole Unger, Lukas van Baal, Jürgen Honegger, Sabrina Giese, Timo Deutschbein, Mario Detomas, Karsten Wrede, Yahya Ahmadipour, Ilonka Kreitschmann-Andermahr

**Affiliations:** 1 Department of Neurosurgery and Spine Surgery, Member of ENDO-ERN, University Hospital Essen, University of Duisburg-Essen, Essen, Germany; 2 German Cancer Consortium (DKTK) Partner Site, University Hospital Essen, University of Duisburg-Essen, Essen, Germany; 3 Center for Translational Neuro- & Behavioral Sciences (C-TNBS), University of Duisburg-Essen, Essen, Germany; 4 Institute of Medical Psychology and Behavioral Immunobiology, University Hospital Essen, University of Duisburg-Essen, Essen, Germany; 5 Department of Endocrinology, Diabetes and Metabolism, Member of ENDO-ERN, University Hospital Essen, University of Duisburg-Essen, Essen, Germany; 6 Department of Neurosurgery, Eberhard-Karls-University Tübingen, Tübingen, Germany; 7 Medicover Oldenburg MVZ, Oldenburg, Germany; 8 Department of Internal Medicine I, Division of Endocrinology and Diabetes, University Hospital Würzburg, Würzburg, Germany

**Keywords:** Acromegaly, Pain, Physical and mental QoL

## Abstract

**Objective:**

Headache and joint pain have been described as relevant factors for the disease-specific burden of acromegaly. We aimed to determine whether pain has a differential influence on physical and mental quality of life (QoL) in this patient group.

**Methods:**

124 patients with acromegaly (age 18–79 years, median 59.0) from three tertiary endocrine and/or neurosurgical centers completed self-constructed questionnaires on acromegaly symptoms and pain and the Western Ontario and McMaster Universities Arthritis Index (WOMAC). QoL was assessed with the Short Form (SF)-36.

**Results:**

Overall, 65% (*N* = 81) of patients reported pain attributed to acromegaly, whereas 35% (*N* = 43) did not. These two groups differed in pain (*z* = -5.62, *p* < .001), stiffness (*z* = -4.05, *p* < .001) and physical activity (*z* = -5.91, *p* < .001), and pain during daily activities, with significantly greater impairments in patients reporting pain attributed to acromegaly. This group also exhibited a significantly worse QoL on the physical (z = -5.90, *p* < .001) and mental level (z = -2.68, *p* = .007), and a higher BMI *(z* = -3.16, *p* = .002). A predictive model showed that higher scores in WOMAC pain, any joint pain, higher age, and higher BMI were significantly associated with reduced physical QoL (72% explained variance). In contrast, mental QoL variability was explained to a much lesser extent (9%).

**Conclusion:**

The results show the differential effect of pain on physical and mental QoL, emphasizing that patients with acromegaly require not only routine assessment of pain but also adequate management of pain and associated symptoms.

**Supplementary Information:**

The online version contains supplementary material available at 10.1007/s12020-026-04566-y.

## Introduction

Acromegaly is a chronic endocrine disorder characterized by excessive secretion of growth hormone (GH) resulting in elevated levels of insulin-like growth factor 1 (IGF-1); common symptoms of acromegaly are acral and soft tissue enlargement, gastrointestinal symptoms, sleep apnea and cardiovascular issues [1].

Patients with acromegaly experience a severely impaired quality of life (QoL) that persists even after treatment: in a systematic review of studies including different established measures of QoL, it was determined that QoL remains lower in acromegaly patients compared to the general population, regardless of biochemical control [2]. Maladaptive coping styles have a profound negative impact on psychological/mental QoL in patients with acromegaly [3]. Other predictors of QoL are delay between first presentation of symptoms and diagnosis of acromegaly, body mass index (BMI), number of doctors visited before the diagnosis, and age at diagnosis as well as previous radiotherapy [4].

Moreover, this condition results in a variety of musculoskeletal complications, including joint pain, that significantly impact patients’ QoL [5, 6], in at least one fourth of the patients [7]. Neuropathic-like pain symptoms are relatively rare in controlled acromegaly, occurring in only 9.1% of patients and were observed exclusively in women. An additional 13.6% had undetermined neuropathic-like symptoms [8]. Most joint pain in acromegaly appears to be mechanical in origin, rather than autoimmune causes [9].

Despite higher joint pain scores, use of pain relief therapy and history of joint surgery do not differ significantly between acromegaly patients and those with non-functioning pituitary adenomas. This suggests potential undertreatment of joint symptoms in acromegaly patients [10]. The role of BMI in the association between pain, arthropathy, and QoL is not yet clearly defined [11]. It is reasonable to hypothesize that weight exerts an influence on both pain and QoL.

The construct of physical and mental QoL has been demonstrated to provide a valid reflection of the multifaceted disease aspects associated with acromegaly [12, 13]. Since we have previously shown impaired physical and mental QoL in acromegaly patients, we want to elucidate whether pain has a differential influence on these two dimensions of QoL in this patient group. The study builds on our recently published focus group study on the burden of acromegaly [14] in which headache and joint pain were voiced as burdensome.

We hypothesize that patients with acromegaly who report pain attributable to their condition will exhibit significantly lower mental and physical QoL compared to those without such pain.

Our secondary aim is to identify clinical and demographic factors associated with physical and mental quality of life (QoL) in acromegaly patients, with a focus on the role of pain, joint symptoms, and body composition.

We hypothesize that patients reporting acromegaly-related pain will demonstrate greater impairments in joint function (e.g., stiffness, physical activity) and have a higher BMI compared to patients without pain.

Furthermore, the impact of pain on QoL is expected to be differently reflected in the variables mental and physical QoL.

## Methods

### Sample

The present study was conducted at three major acromegaly treatment centers (Departments of Neurosurgery and Spine Surgery and Endocrinology, Diabetes and Metabolism, University Hospital Essen; Department of Neurosurgery, University Hospital Tübingen; Endocrinological Practice Medicover Oldenburg). Adult patients treated at one of the three centers were recruited for the study by mail if they met the following criteria: Presence of biochemically proven acromegaly due to a GH-secreting pituitary adenoma, sufficient command of the German language to complete a self-report questionnaire, no active psychotic disorder. A total of 328 (38% response rate) eligible patients received a study information sheet, a consent form and questionnaires, sent via mail; therefore, questionnaires were filled out on paper. The study was approved by the ethics committee of the University of Duisburg-Essen (17-7650-BO) and was conducted according to the Declaration of Helsinki. All patients included in the analysis provided written informed consent.

### Questionnaires

#### Demographics and Medical Data

A self-developed, exploratory, patient-reported questionnaire was used to obtain demographic and medical data, as well as information on any pain experienced by the participants. The following data were collected: age, height, weight, sex, date of acromegaly diagnosis, date of last surgery, therapy of the disease, existence of diabetes, degree of disability, disability certificate, and IGF-1 value at the last visit.

All self-developed questionnaires can be found in Supplementary Material.

#### Assessment of Pain

For the purpose of the study, an ad hoc pain questionnaire for acromegaly patients was created, based on insights gained from our experience in daily patient interactions and the results of a focus group analysis on the impact of acromegaly on daily life [14].

Questions to be answered by all participants pertained to presence of pain in different situations and at different times regardless of whether or not this pain was attributed to acromegaly (at work, in certain lying or sitting conditions, in stressful situations, after injecting medications, pain every day, avoiding activities to avoid pain).

Additionally, the Western Ontario and McMaster Universities Arthritis Index (WOMAC) [15, 16] was used as an established pain questionnaire. The WOMAC is a multidimensional, self-administered health status instrument with 24 items to assess physical function, pain and stiffness in patients with osteoarthritis in hip and knees. Each of the three dimensions can have a total score of 0–10, with higher scores reflecting greater impairment.

Another measure contained an introductory question, querying whether the participants suffered of any pain, attributed to acromegaly. Only if answered with “yes”, the participants were asked to mark the location(s) of their pain on a pain location diagram. These locations were clustered by the investigators into the categories: “headache”, “joint pain” and “any other pain”.

Pain was assessed not only as a general symptom but also in relation to acromegaly-specific triggers (e.g., subcutaneous injections), anatomical distribution, and attribution to the disease.

#### Assessment of QoL

QoL was assessed with the Short Form Health Survey (SF-36) [17].The SF-36 consists of 36 items measuring health-related QoL in the last four weeks before the investigation. It covers 8 dimensions (*vitality*,* physical functioning*,* bodily pain*,* general health perceptions*,* physical role functioning*,* emotional role functioning*,* social role functioning*, and *mental health*), which can be combined to compute a physical component summary score (SF-36 PCS) and a mental component summary score (SF-36 MCS). PCS and MCS are calculated by standardizing the eight scale scores to a z-score, and then summing these z-scores after they are multiplied by specific factor-scoring coefficients. The transformed subscales are scaled from 0 to 100; the transformed summary scores have a mean of 50 and a standard deviation (SD) of 10 (T-scores). Higher scores represent a better QoL. The standardization allows for comparison with the age- and sex-matched reference values of the German normative population [18]. The standardized deviation scores (SDS) were also recorded. A score > -2 SDS below mean indicates a severely impaired QoL, and a score between − 1 and − 2 SDS indicated an impaired QoL.

The major advantages of the SF-36 over disease-specific measures include its factor structure, which provides reliable and independent measurements of mental and physical QoL, and its very large normative samples. The standard itself acts as a kind of “natural” control group, rendering the need for a separate control group unnecessary.

### Statistical analysis

Data were analyzed using IBM Corp. Released 2023. IBM SPSS Statistics for Windows, Version 29.0.2.0 Armonk, NY: IBM Corp. Because data did not meet the assumption of normality, which was assessed using the Shapiro-Wilk-test, non-parametric statistical analyses was performed. Differences between patients of the three treatment centers as well as BMI-groups were calculated with the Kruskal-Wallis-H-test (and post-hoc with the Mann-Whitney-U-test) or with the chi²-test, respectively, to evaluate potential site effects. To control for multiple comparison, levels of significance were Bonferroni-corrected. Differences between patients with and without acromegaly-attributed pain were analyzed using Mann-Whitney-U-test (rank sums: U-values and test statistic: Z-scores) or chi²-test, respectively. To assess the potential influence of pain and other factors on QoL stepwise multiple-regression analyses were performed on the entire sample; violation of the normality assumption of linear regression models with large sample sizes is tolerable [19]. Variables representing aspects of pain were included in the regression model as potential predictors. To limit the number of variables and due to high intercorrelations between the different variables describing aspects of pain, we decided to use the following variables: WOMAC pain, any headache, any joint pain and any other pain. We also included age, BMI and disease duration to include socio-demographic and disease characteristics as influencing factors. Predictors were integrated into the model, if the value falls below *p* = .05, and removed again if *p* = .10 is exceeded. The level of significance was set at *p* < .05 (0.05 / 3 after Bonferroni-correction). All data are expressed as median (IQR). All proportions are reported in valid percent. Missing values were regarded as missing at random and were deleted casewise from the analysis.

## Results

### Description of study sample

A total of 124 patients (44.6% females) aged 18–79 years, with a median of 59.0 (49.0; 65.0) years were included in the analysis. The median BMI was 28.7 (25.8; 32.2) kg/m^2^ and ranged from 18.7 to 46.7 kg/m^2^. All patients suffered from clinically and biochemically proven acromegaly and had a median duration of disease since diagnosis of 10.0 (4; 17) years with a range of 1–39 years. A total of 118 patients (95.2%) had undergone pituitary surgery. Median time since last surgery was 10 (5; 16.3) years, ranging from 1 to 41 years. At their last consultation, 81.8% of the patients had a normalized IGF-1 value. 95.2% of the patients had been operated on a GH-secreting pituitary adenoma, 18.0% had received radiation therapy. 29.5% of all patients had gonadotropic insufficiency, 59.1% of them replaced. 19.2% had corticotropic insufficiency, all of them replaced. 28.2% had thyreotropic insufficiency, all of them replaced. 3.8% of the patients had arginine vasopressin deficiency, all of them treated. 55.6% had official degree of disability and 8.4% were on sick leave. 51.7% were employed (full- or part-time). The patients of the three centers did not differ in most sociodemographic characteristics (age (*H*(2) = 2.45, *p* = .295), sex (Chi²(2) = 0.84, *p* = .658)), except for BMI (*H*(2) = 6.59, *p* = .037), i.e. patients in Essen had a higher BMI (30.5 (27.5; 33.1)) than patients in Tübingen (26.5 (24.5; 30.8); *U* = 583.00, *z* = -2.66, *p* = .008).

### Pain symptoms

Overall, 65.32% (*N* = 81) of the patients stated to have pain which they attributed to their acromegaly, whereas 34.68% (*N* = 43) did not report acromegaly attributed pain. 69.14% of the former group of patients experienced headache, 80.25% joint pain and 60.49% pain in any other location, attributed to their illness.

Participants had a median WOMAC score of 2.7 (1.0; 5.1) for pain, 3.3 (1.5; 6.1) for stiffness and 2.5 (1.0; 4.9) for physical function. 54.84% of patients reported pain at work, 58.07% reported pain when lying or sitting, 41.94% reported pain during stress, 22.58% reported pain after injecting medication, 59.68% reported daily pain and 42.74% stated to avoid some activities to prevent pain.

The two groups (patients with and without acromegaly-attributed pain) did not differ in age (*U* = 1601.00, *z* = -0.63, *p* = .528), sex (*Chi²*(1) = 0.207, *p* = .649), or employment (*Chi²*(1) = 0.01, *p* = .909), but in BMI (*U* = 1123.50, *z* = -3.16, *p* = .002). Regarding clinical characteristics, they differed in the intake of acromegaly-specific medication (*Chi²*(1) = 6.92, *p* = .009), and degree of disability (*Chi²*(1) = 16.14, *p* = < 0.001), but not in radiation therapy (*Chi²*(1) = 0.61, *p* = .435) or normalization of IGF-1 (*Chi²*(1) = 2.58, *p* = .108). They did, however, differ significantly in the pain (*U* = 487.00, *z* = -5.62, *p* < .001), stiffness (*U* = 738.50, *z* = -4.05, *p* < .001) and physical activity (*U* = 439.00, *z* = -5.91, *p* < .001) subscales of the WOMAC, with patients with perceived pain attributed to acromegaly exhibiting greater impairments (confer to Table 1). Further, more of these patients reported having pain at work (*Chi²*(1) = 49.63, *p* < .001), when lying or sitting (*Chi²*(1) = 37.28, *p* < .001), in stressful situations (*Chi²*(1) = 28.79, *p* < .001), after injecting medication (*Chi²*(1) = 9.17, *p* = .002), in feeling pain every day (*Chi²*(1) = 51.52, *p* < .001) as well as avoiding activities to prevent pain (*Chi²*(1) = 26.04, *p* < .001; compare Table 1).

**Table 1:** Sociodemographic and clinical characteristics, impairments and QoL of patients with and without perceived pain attributed to acromegaly, displayed are median (IQR) or N and valid percent; the categories “headache”, “joint pain” or “any other pain” were only applicable for patients that attributed their pain to acromegaly

	Total sample (*n* = 124)	Patients with acromegaly-attributed pain (*n* = 81)	Patients without acromegaly-attributed pain (*n* = 43)	*p*-value
**Sociodemographic characteristics**				
Age	59.00 (49.00; 65,00)	59.50 (52.00; 64.00)	56.00 (46.00; 66.00)	0.528
Sex (male/female)	55.37% / 44.63%	53.85% / 46.15%	58.14% / 41.86%	0.649
BMI	28.69 (25.76; 32.21)	29.86 (26.08; 33.16)	26.85 (23.78; 30.10)	**0.002**
Employed (yes/no)	51.70% / 48.30%	51.30% / 48.70%	52.40% / 47.60%	0.909
**Clinical characteristics**				
Current acromegaly medication (yes/no)	59.0% / 41.0%	67.5% / 32.5%	42.9% / 57.1%	**0.009**
Radiation therapy (yes/no)	18.0% / 82.0%	20.0% / 80.0%	14.3% / 85.7%	0.435
Degree of disability (yes/no)	55.60% / 44.40%	68.40% / 31.60%	28.90% / 71.10%	**< 0.001**
IGF-1 normalized (yes/no)	81.80% / 18.20%	76.90% / 23.10%	92.00% / 8.00%	0.108
**Impairments**				
Headache	56 (45.16%)	56 (69.14%)	0.00%	**< 0.001**
Joint pain	65 (52.42%)	65 (80.25%)	0.00%	**< 0.001**
Any other pain	49 (39.52%)	49 (60.49%)	0.00%	**< 0.001**
WOMAC pain	2.70 (1.00; 5.10)	3.80 (2.20; 6.40)	1.00 (0.00; 2.00)	**< 0.001**
WOMAC stiffness	3.25 (1.50; 6.13)	5.00 (2.00; 7.00)	1.50 (1.00; 3.50)	**< 0.001**
WOMAC physical activity	2.47 (1.00; 4.85)	3.88 (2.18; 5.71)	1.00 (0.35; 1.35)	**< 0.001**
pain at work	68 (54.84%)	63 (77.78%)	5 (11.63%)	**< 0.001**
pain in lying / sitting position	72 (58.07%)	63 (77.78%)	9 (20.93%)	**< 0.001**
pain in stressful situations	52 (41.94%)	48 (59.26%)	4 (9.30%)	**< 0.001**
pain after injection of medication	28 (22.58%)	25 (30.86%)	3 (6.98%)	**0.002**
pain every day	74 (59.68%)	67 (82.72%)	7 (16.28%)	**< 0.001**
avoiding activities	53 (42.74%)	48 (59.26%)	5 (11.63%)	**< 0.001**
**Quality of Life**				
SF-36 physical component	40.24 (27.51; 51.62)	33.36 (25.41; 44.39)	52.06 (46.06; 56.07)	**< 0.001**
SF-36 mental component	45.15 (33.59; 54.16)	41.23 (32.51; 52.74)	52.27 (40.69; 55.61)	**0.007**
SF-36 Physical Functioning	70.00 (35.00; 90.00)	55.00 (30.00; 75.00)	95.00 (80.00; 100.00)	**< 0.001**
SF-36 Physical Role Functioning	50.00 (0.00; 100.00)	25.00 (0.00; 75.00)	100.00 (75.00; 100.00)	**< 0.001**
SF-36 Physical Pain	52.00 (32.00; 84.00)	41.00 (22.00; 62.00)	100.00 (72.00; 100.00)	**< 0.001**
SF-36 General Health Perception	47.00 (35.00; 62.00)	42.88 (25.50; 55.00)	67.00 (47.00; 77.00)	**< 0.001**
SF-36 Vitality	45.00 (30.00; 58.75)	35.00 (20.00; 50.00)	55.00 (40.00; 65.00)	**< 0.001**
SF-36 Social Functioning	75.00 (50.00; 100.00)	62.50 (43.75; 87.50)	87.50 (71.88; 100.00)	**< 0.001**
SF-36 Emotional Role Functioning	66.67 (0.00; 100.00)	33.33 (0.00; 100.00)	100.00 (66.67; 100.00)	**0.002**
SF-36 Mental Well-Being	64.00 (44.00; 76.00)	60.00 (40.00; 72.00)	76.00 (56.00; 84.00)	**< 0.001**

### BMI

Twenty-three patients were normal weight (BMI 18.5–24.9) and had a median BMI of 23.6 (23.2; 24.3), 50 patients were overweight (BMI 25-29.9) with a median BMI of 26.9 (25.9; 28.6) and 50 patients were obese (BMI ≥ 30) with a median BMI of 33.5 (31.4; 37.4). 78.0% of obese patients attributed their pain to acromegaly, which was significantly more (*Chi²*(1) = 6.65, *p* = .010) than in normal weight patients (47.8% ), but did not differ significantly (*Chi²*(1) = 3.79, *p* = .052) from overweight patients (60.0%), who in turn did not differ significantly (*Chi²*(1) = 0.95, *p* = .330) from normal weight patients. However, no differences were found regarding experienced headache, joint pain and pain in any other location, attributed to the illness (all *Chi²*(2) ≤ 3.81, *p* ≥ .149). BMI-groups differed significantly in WOMAC scores, with obese patients reporting greater impairments than patients of normal weight (WOMAC pain: *U* = 208.50, *z* = -3.59, *p* < .001; WOMAC stiffness: *U* = 274.50, *z* = -2.69, *p* = .007; WOMAC physical activity: *U* = 209.00, *z* = -3.58, *p* < .001) as well as overweight patients in terms of physical activity (*U* = 669.50, *z* = -2.61, *p* = .009; all other *U* ≤ 714.50, z ≤|-2.24|, *p* ≥ .025).

### Quality of life

Patients had an overall median score of 40.2 (27.5; 51.6) on the SF-36 PCS and a median score of 45.2 (33.6; 54.2) on the SF-36 MCS, which is within one SD compared to the healthy population. Patients with pain attributed to acromegaly had lower QoL than patients without pain attributed to acromegaly at both the physical (U = 510.00, z = -5.90, *p* < .001) and psychological (U = 1072.00, z = -2.68, *p* = .007; see Table 1) levels. In the predictive model for the physical component of QoL, which included *all* patients, WOMAC pain, any joint pain, age and BMI were found to be significantly associated with reduced physical QoL (F(4, 92) = 64.08, *p* < .001). This model explained 72.4% of the variance. In contrast, the variance in mental QoL was explained to a much lesser extent, namely only 8.8%. The corresponding model showed that only WOMAC pain was significantly related to reduced mental QoL (F(1, 97) = 10.43, *p* = .002; see Table 2).

**Table 2:** Results of Stepwise multiple-regression analyses with physical and mental QoL (SF-36) as criterion

Physical QoL	B	SE	β	*p*-value	*R* ^2^
Constant	83.80 (73.65, 93.95)	5.11	–	< 0.001	
WOMAC pain	-2.72 (-3.40, -2.04)	0.34	− 0.53	< 0.001	0.594
Age	− 0.26 (-0.37, − 0.15)	0.06	− 0.26	< 0.001	0.649
Any joint pain	-6.39 (-9.68, -3.11)	1.65	− 0.24	< 0.001	0.685
BMI	− 0.54 (-0.82, − 0.26)	0.14	− 0.22	< 0.001	0.724
**Mental QoL**					
Constant	49.47 (45.94, 53.01)	1.78	–	< 0.001	
WOMAC pain	-1.38 (-2.23, − 0.53)	0.43	− 0.31	0.002	0.088

*B* regression coefficient (95%CI), *SE* standard error, *β* standardized regression coefficient, *R*^*2*^ coefficient of determination of each step of the regression model*.*

## Discussion

This study highlights the central role of pain as a key determinant of physical health-related quality of life (QoL) in patients with acromegaly. Our results show that patients with pain attributed to acromegaly report significantly greater impairment in physical QoL compared to patients without acromegaly attributed pain. Regression analysis revealed that pain (acromegaly attributed or not), together with clinical characteristics such as age and BMI, explained a substantial 72.4% of the variance in physical QoL, but only 8.8% of the variance in mental QoL. This strong association underscores that pain is not only a common symptom, but also an important organic factor that directly limits physical functioning and daily activities in this patient population.

Impairment of QoL is common, even with controlled acromegaly [2, 20]. In this study, more than 80% of patients reported a normalized IGF-1 level at the last visit, with limited interpretability due to missing values. Median QoL scores did not deviate from the normal population with respect to the mental component, but were at the lower limit of impairment with respect to the physical component, considering normative values. This suggests that pain, often resulting from irreversible musculoskeletal changes such as arthropathy and joint degeneration, persists as an organic consequence of the disease and continues to limit physical functioning.

Mental QoL as assessed with the SF-36, was, on average, within the statistical normal range of one standard deviation and higher compared to the physical QoL in the present cohort. Time since diagnosis and surgery in this cohort were both an average of ten years ago, and it is likely, that participants had adjusted to some of the impairments within such a long time period. A previous study found that worse mental QoL was associated with having received radiotherapy [4]. Therefore, another explanation for the on average high mental QoL scores in our study could be that only about one-fifth of the patients had received radiotherapy. On the other hand, the results regarding the influence of radiotherapy on QoL are controversial in pituitary tumors including acromegaly [21]. The mean BMI in the present cohort was classified as overweight [22]. BMI is associated with osteoarthritis and pain in general [23]. This means that acromegalic patients with higher BMI may be at risk for experiencing more pain regardless of their underlying disease. Indeed, obese acromegaly patients in our study perceived pain differently than normal-weight and overweight study participants in some, but not all, pain measures.

Although BMI provides a straightforward, population-level estimate of body fat based on weight and height (kg/m²), it lacks specificity in assessing body composition. The BMI is a measure that fails to differentiate between fat mass (FM), lean mass (LM), and fat distribution. This limitation can result in misclassification, particularly among individuals with high muscle mass (e.g., athletes) or low muscle mass (e.g., the elderly or those with sarcopenia) [24].

While BMI remains a useful screening tool, body composition assessment provides a more nuanced, clinically relevant understanding of health risks and metabolic health, particularly in diverse populations and chronic disease contexts, such as acromegaly.

Pain was assessed using patient-reported measures, which are essential for capturing the lived experience of chronic symptoms. The presence of comorbidities may have influenced the assessment of pain. Common comorbidities in acromegaly are cardiac dysfunction, fatigue, sleep apnea, depression; the presence of musculoskeletal pain can be symptom of comorbidities like arthralgia [25]. Additionally, pain can be estimated differently when psychiatric comorbidities are present. Depression, QoL and arthropathy have been shown to interact [26], with possible higher pain scoring of depressive patients or, reversely, pain enhancing mood symptoms and having a negative impact on QoL.

The use of the WOMAC pain scale in our study specifically captured musculoskeletal pain, which is known to be a prominent and disabling feature of acromegaly mediating reduced physical and mental QoL [27]. The mean stiffness and pain scores for the entire cohort in the present study were similar to that in other studies [11, 28, 29], whereas the physical activity score was lower. This suggests that our patients experienced fewer functional limitations related to osteoarthritis. The WOMAC scores of patients without acromegaly-related pain were similar to those of a healthy cohort [28]. Patients who attributed their pain to acromegaly reported higher pain scores in all domains, reflecting the ongoing organic burden of the disease on the musculoskeletal system. This is consistent with the finding that fatigue/muscle weakness and joint pain/arthritis are the most commonly reported and bothersome symptoms in acromegaly [30]. Compared to nonfunctioning pituitary adenomas, acromegaly patients also had higher joint pain scores [31]. Arthropathy as a result of excess growth hormone appears to trigger an inflammatory response that affects all joint tissues [32, 33].

The pervasive nature of pain in acromegaly is also reflected in the high proportion of patients reporting daily pain, pain while working, and pain while lying down or sitting. Over 40% of participants reported avoiding activities due to pain, directly linking pain to physical inactivity and functional limitations. These findings highlight the organic impact of pain as a barrier to mobility, participation in daily activities, and overall physical well-being. While psychological factors may also play a role, our data underscore that the primary limitation to physical QoL in this cohort is driven by the organic consequences of pain and musculoskeletal impairment. Interestingly, radiographic progression of arthropathy was not strictly related to clinical progression in a previous study [34], underscoring the need for detailed clinical assessment in the management of patients with acromegaly.

Previous research using an acromegaly specific QoL tool, the AcroQoL, confirmed that pain symptom severity predicts QoL in acromegaly [11, 26]. The distinction between physical and mental QOL, as measured by the SF-36, can provide further insight into patient burden [35]. Although pain emerged as the dominant factor affecting physical QoL, other clinical variables such as age and BMI also contributed to overall impairment. Nevertheless, the overwhelming influence of pain on physical QoL, as demonstrated by our regression analysis, suggests that pain perception should be a primary focus in the management of acromegaly-related QoL deficits.

Mental QoL was only influenced by WOMAC pain and to a much lesser extent. There may be several explanations for this finding. Mental QoL is known to be influenced by the number of doctors seen, previous radiotherapy, age at study entry and diagnostic delay, as described above [4, 35]. These are not exactly factors that could be associated with the experience of pain.

From the present results, we conclude that care of patients with acromegaly necessitates assessment of pain measures and management of pain symptoms, as well as teaching helpful coping strategies and weight control. Coping strategies may help to deal with the disease and thereby influence management of pain symptoms indirectly. Weight control could add to the effects in directly reducing bodily pain.

The present work is a multicenter study with a large sample size of acromegalic patients with robust statistical results. The study has several limitations. The cross-sectional design precludes causal inference, and collection of medical data was based on patient self-report, which may introduce bias. In addition, we did not assess psychological comorbidities, which may interact with organic factors to influence overall QoL. The influence of medical treatment and IGF-1 levels was not analyzed due to the patient-reported nature of all parameters, which resulted in a high number of missing values. Future research should employ longitudinal designs, incorporate objective measures of musculoskeletal impairment and disease activity parameters, and evaluate the effectiveness of targeted and person-centered pain interventions in improving physical QoL. Multimodal treatment approaches with medications and non-pharmacological methods like cognitive behavioral therapy [36, 37] should be implemented.

In conclusion, our findings underscore the central role of pain – especially of organic, musculoskeletal origin – as a primary cause of impaired physical QoL in patients with acromegaly. Effective assessment and management of pain should be prioritized to address the significant physical limitations experienced by this patient population. In future studies, we need to investigate the use of pain therapies and the unmet need for pain management in patients with controlled acromegaly, as well as gather feedback on acromegaly patients’ interest in new forms of therapy to reduce pain and improve QoL. Given the strong link between pain and physical QoL, comprehensive pain assessment and targeted management of musculoskeletal pain are essential components of care for patients with acromegaly. While psychosocial factors undoubtedly influence pain perception and QoL, the presence of structural and metabolic abnormalities in acromegaly—such as joint degeneration, soft tissue overgrowth, and hormonal imbalances—provides a clear organic basis for many pain symptoms. Therefore, a comprehensive management strategy should integrate both organic and psychosocial interventions.

## Supplementary Information

Below is the link to the electronic supplementary material.

Supplementary Material 1

## Data Availability

Any data not published within the article will be shared in an anonymized manner at request from any qualified investigator.
