# Supplementary material for: Impact of pain on quality of life (QoL) in patients with acromegaly: profound differences in physical and mental aspects of QoL
Source: Endocrine. 2026 Mar 9;91(1):99. doi: 10.1007/s12020-026-04566-y (PMC12971745; doi:10.1007/s12020-026-04566-y)
Supplement: Supplementary file 1 — Supplementary Material 1 [file 12020_2026_4566_MOESM1_ESM.pdf]

Pro-Questionnaire Acromegaly

Dear patient,
Below you will find some questions about your living situation, the course of your illness, and your support needs in connection with your acromegaly. We ask you to answer all questions as completely as possible and not to omit any.
Thank you for your cooperation!

Personal Data

ID code ☐	Today's date ☐
Age ☐	Gender<br>☐ male<br>☐ female

Place of residence

Location ☐	Postal code ☐

CURRENT life situation

**I live:**<br>☐ Alone<br>☐ Alone with children<br>☐ With my life partner<br>☐ With my partner and children<br>☐ In a shared apartment<br>☐ At my parents' house<br>☐ Supervised	**Marital status :**<br>☐ Single<br>☐ Married<br>☐ Divorced<br>☐ Widowed

Training

**Highest school degree:**<br>☐ None<br>☐ Lower secondary school leaving certificate<br>☐ Higher secondary school leaving certificate<br>☐ Vocational baccalaureate diploma<br>☐ High school diploma	**Do you have a completed vocational training?**<br>☐ Yes<br>☐ No<br>**Do you have an university degree?**<br>☐ Yes<br>☐ No

CURRENT work situation

Profession

If you are employed, are you currently on sick leave?

☐ Yes , since ______/______/________(day/month/year)

☐ No

Work situation

☐ Full-time

☐ Part-time

☐ Unemployed

☐ Housewife/ Househusband

☐ Disability pension

☐ Early retirement

☐ Pension

If you are not yet retired, have you submitted a pension application?

☐ Yes , on ______/______/________(day/month/year)

☐ No

Do you have a recognized degree of disability (GdB) (e.g., from the Office for Supply Affairs)?

☐ Yes . If so, what is the degree of disability (GdB) ? ________%

Has a higher classification been requested? ☐ Yes ☐ No

☐ No. If no, have you submitted an application? ☐ Yes ☐ No

Health insurance

How are you insured?

☐ Statutory health insurance

☐ Private health insurance

- ☐ Without assistance
- ☐ With assistance

Physical data

Size (m)

Weight (kg)

Do you smoke ? If so, how much ?

☐ Yes , _______________ Cigarettes /day

☐ No

Treatment of acromegaly

Have you had surgery on your pituitary gland (hypophysis) due to acromegaly?

☐ Yes ☐ No

If so, how often and when?

1. Surgery When? : _______ /_________ (Month/Year)

2. Surgery When ? : _______ /_________ (Month/Year)

3. Surgery When ? : _______ /_________ (Month/Year)

Are you currently taking medication for acromegaly?

☐ No ☐ Yes

**If so, which ones?**	**Yes**	**No**
Somatostatin analogues	☐	☐
Dopamine agonists	☐	☐
Pegvisomant	☐	☐

Have you ever received radiation therapy to your head ?

☐ No ☐ Yes If yes, when ? **from** (Month/Year): _______ /_______

until (Month/Year): _______ /________

Current symptoms

Are you currently experiencing any of the following symptoms? (Multiple answers possible)

☐ Headache

☐ Sweat

☐ Joint problems

☐ Swelling

☐ Miscellaneous: ____

____

Current symptoms

Are you currently experiencing pain that you suspect is caused by acromegaly?

☐ No ☐ Yes

If so, please mark on the body diagram where you experience pain.

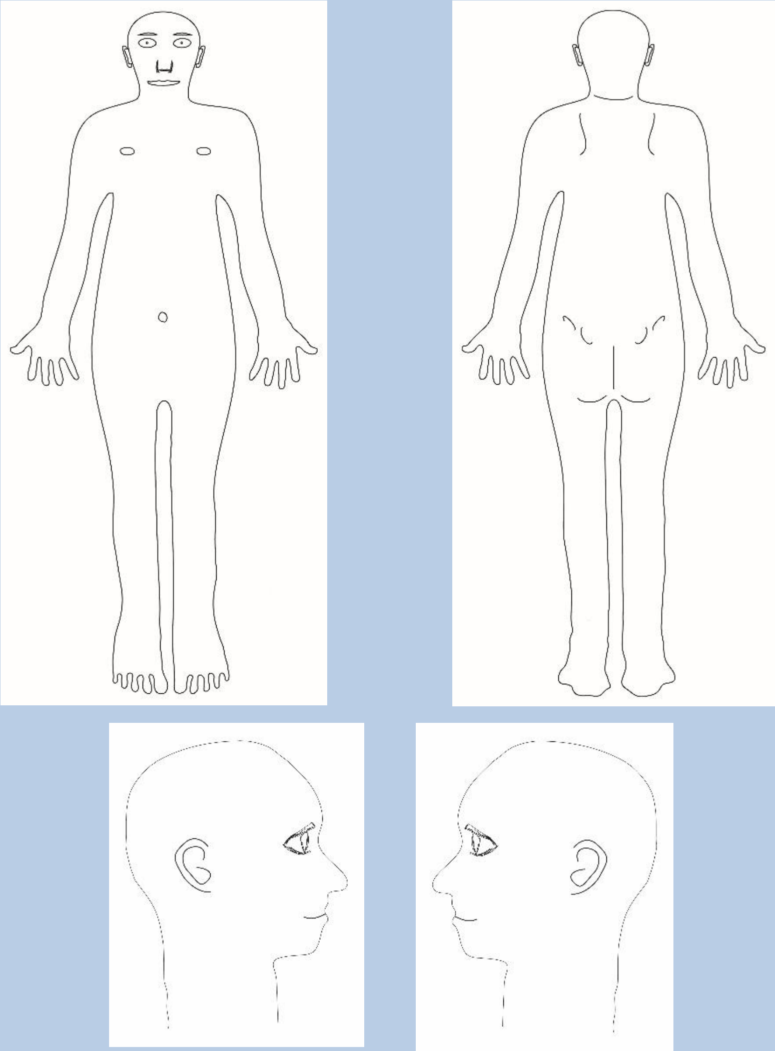

Please describe your pain in your own words:

____

____

____

____

____

____

____

Pain

How much do the consequences of acromegaly listed below affect you?

(0) not at all (1) a little (2) quite (3) strongly (4) very strongly ◯ not applicable

Pain						
1. I experience pain at work.	0	1	2	3	4	◯
2. I experience pain in certain sitting or lying positions.	0	1	2	3	4	◯
3. I'm in pain every day.	0	1	2	3	4	◯
4. I experience pain in stressful situations.	0	1	2	3	4	◯
5. I have pain after the injection of the medication.	0	1	2	3	4	◯
6. I avoid certain activities to avoid getting pain.	0	1	2	3	4	◯
